# Rhynchophylline Protects Cultured Rat Neurons against Methamphetamine Cytotoxicity

**DOI:** 10.1155/2012/636091

**Published:** 2012-03-01

**Authors:** Dan Dan Xu, Robin Hoeven, Rong Rong, William Chi-Shing Cho

**Affiliations:** ^1^Faculty of Science, The Chinese University of Hong Kong, Hong Kong; ^2^Jiujianpeng Technology R&D Center, Linyi, China; ^3^Faculty of Science, Katholieke Universiteit, Leuven, Belgium; ^4^School of Pharmacy, Shandong University of Traditional Chinese Medicine, Jinan, China; ^5^Department of Clinical Oncology, Queen Elizabeth Hospital, Hong Kong

## Abstract

Rhynchophylline (Rhy) is an active component isolated from species of the genus *Uncaria* which has been used for the treatment of ailments to the central nervous system in traditional Chinese medicine. Besides acting as a calcium channel blocker, Rhy was also reported to be able to protect against glutamate-induced neuronal death. We thus hypothesize that Rhy may have neuroprotective activity against methamphetamine (MA). The primary neurons were cultured directly from the cerebral cortex of neonatal rats, acting as *in vitro* model in the present study. The neurotoxicity of MA and the protective effect of Rhy were evaluated by MTT assay. The effects of MA, Rhy or their combination on intracellular free calcium concentration ([Ca^2+^]_*i*_) were determined in individual neocortical neurons by the Fluo-3/AM tracing method. The MTT assay demonstrated that MA has a dose-dependent neurotoxicity in neuronal cultures. The addition of Rhy prior to the exposure to MA prevented neuronal death. Time course studies with the Fluo-3/AM probe showed that Rhy significantly decreased neuronal [Ca^2+^]_*i*_ which was elevated by the exposure to MA. Our results suggested that Rhy can protect the neuronal cultures against MA exposure and promptly attenuate intracellular calcium overload triggered by MA challenge. This is the first report demonstrating an inhibitory effect of Rhy against MA impairment in cultured neurons *in vitro*.

## 1. Introduction

**Figure 1:**
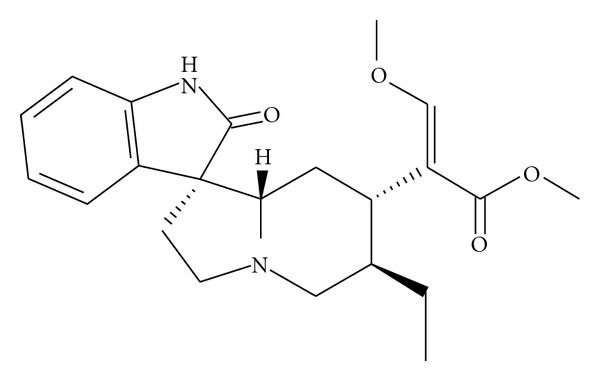
Chemical structure of rhynchophylline.

Rhynchophylline (Rhy) is a major tetracyclic oxindole alkaloid (the chemical structure of Rhy is shown in Figure 1), originally isolated from species of the genus *Uncaria* which is widely used in traditional Chinese medicine prescribed mainly to treat ailments to the central nervous and cardiovascular systems, such as lightheadedness, convulsions, numbness, and hypertension [1].

In the cardiovascular system, it is well documented that the vasodilative effect of Rhy is mainly due to the dysfunction of Ca^2+^ transport, including influx of extracellular calcium and release of intracellular calcium by blocking the voltage-dependent calcium channel and the receptor-regulation calcium channel [2]. There are records that an extraction with methanol from *Uncaria rhynchophylla* exhibits a significant neuroprotective effect *in vitro*, in which Rhy appears to be the active pharmacological component [3]. Further research on rat cerebellar granule cells showed Rhy to be able to protect against glutamate-induced neuronal death [4]. Moreover, Rhy is thought to have calcium channel blocking functions, which can further explain the neuroprotective functions. Calcium channel blockage can potentially have a protective effect because of the positive relation between increased calcium influx and cell death [1]. For this reason, we expect Rhy to have neuroprotective functions for the central nervous system.

However, the mechanistic study for Rhy is limited, especially for the neural impairment induced by drug abuse. Methamphetamine (MA) is a commonly abused psychostimulant in the world, which leads to severely neurodegenerative changes in the human brain [5]. It primarily acts on dopamine transporter (DAT) and vesicular monoamine transporter-2 (VMAT-2), which when dysfunctioning result in high extracellular dopamine concentrations and subsequent neurotoxicity [6]. Repeated administration of MA has neurotoxic properties, which are caused by an intracellular calcium overload [7]. Several *in vitro* and *in vivo* studies suggest that MA might cause cell death *via* a process that resembles apoptosis *in vitro* and *in vivo* [8]. Calcium signaling plays an important role in apoptosis, which mediates several important steps in the apoptotic downstream pathway. The inhibition of Ca^2+^ influx in a rat cerebellum neural cell line (R2) was reported recently to be able to attenuate the neurotoxicity of MA [9]. We thus hypothesize that Rhy may have neuroprotective activity against MA-induced intracellular calcium overload. In the present study, the neurotoxicity of MA was examined and then saved by the potential calcium antagonist Rhy in the primary rat neuronal cells in culture.

## 2. Materials and Methods

### 2.1. Preparation of Drug

Rhy was extracted from plants of *Uncaria* species by Matsuura Ykugyo Co Ltd (Japan) at purity of 99.7%. It was first dissolved in dimethyl sulphoxide (DMSO; Sigma, USA) and then diluted in the sterile cell culture medium. The final concentration of DMSO was <0.1%. MA hydrochloride was acquired from the National Laboratory on Narcotic Drugs (PR China).

### 2.2. Primary Cell Culture

Experiments were performed in accordance with the recommendations from the Guide for Animal Experimentation of The Chinese University of Hong Kong in the care and use of experimental animals. Neonatal Wister rats (postnatal day 1) were used for the primary neuronal cell culture. Rats were sacrificed by decapitation and brain regions of the cortex were dissected on ice. The tissues were put into an eppendorf with 1 mL minimal essential medium (MEM; Gibco, USA) with 1% penicillin/streptomycin (PSN; Gibco) in ice. Then the brain tissues were chopped using a razor blade in MEM. The chopped tissues were then treated with prewarmed trypsin/EDTA solution with 1% PSN for 20 minutes at 37°C in a 5% CO_2_ incubator and agitated with a dropper every 5 minutes. MEM solution with 10% fetal bovine serum (Gibco) and 1% PSN was added to stop the trypsinization. Then the solution was centrifuged at 1,300 rpm for 10 minutes. The supernatant was removed and the cells at the bottom were suspended in MEM solution and centrifuged again. Finally, the supernatant was removed and the cells inside were resuspended in the complete culture medium, composed of Neurobasal-A-Medium, 10% fetal bovine serum, 1% PSN, and 0.1% 50 × B27 supplement (Gibco). The neurons were then seeded on poly-L-lysine (Sigma) precoated 96-well microplates for MTT (3-(4,5-dimethylthiazol-2-yl)-2, 5-diphenyltetrazolium bromide) assay, or seeded on glass bottom culture dishes (MatTek Corporation, USA) for intracellular calcium recording under confocal microscope. The cultures were maintained at 37°C in a 5% CO_2_ humidified incubator. We used serum-free medium (Neurobasal-A-Medium, 1% PSN, 0.1% 50 × B27 supplement; Gibco), complemented with 1 *μ*M cytosine arabinoside (Sigma), to gradually replace complete medium. The addition of a selective DNA synthesis inhibitor (cytosine arabinoside) in culture eliminated proliferative glial cells allowed adequate numbers of neurons to selectively survive.

### 2.3. Cell Viability Assay

Cell viability with MA treatment was determined by measuring the mitochondrial dehydrogenase activity using MTT assay in triplicate. The cortical neurons, with a seeding number of 10^4^ cells per well in a 96-well microplate, were cultured for 6 days before use. The cells were incubated with MA at serial concentrations (0, 25, 50, 100, 200, and 400 *μ*M) for 48 hours at 37°C. After 44 hours, 10 *μ*L MTT solution (1 g/L; Sigma) was added into each well, and the microplate was incubated for 4 hours. Formazan crystals, produced by mitochondrial dehydrogenase activity in viable neurons, were dissolved by addition of 150 *μ*L DMSO on a shaker at room temperature. Absorbances were read at 570 nm using a microplate reader.

Vehicle controls were set up in parallel to offset the background during process. Each test was performed in triplicate in the 96-well microplates. The experiment was repeated for 3 times. Cytotoxic effect was analyzed by generating dose-response curves as graphs of viable cells (*y*-axis) against the concentration of MA (*x*-axis) with assistance of Prism software (Graph Pad Software Inc, USA). The viable cells were measured in percentage with respect to the following formula:

(1) $$\text{Viability}=\frac{{\text{A}}_{\text{MA-treated}}-A_{\text{vehicle}\;\text{control}}}{A_{\text{normal}\;\text{neurons}}-A_{\text{vehicle}\;\text{control}}}\times 100\%$$

### 2.4. Microscopy Observation

The morphological changes in neurons were monitored under an inverted phase-contrast microscope before and after MA treatment.

### 2.5. Measurement of Cell Viability in the Combination Study of MA and Rhy

In the combination study, MA was added 10 minutes prior to Rhy. The cultures were then left to incubate for 48 hours. Cell viability was determined by MTT assay as previously described. Each test was performed in triplicate in 96-well microplates. Absorbances of resulting formazan crystals were measured at 570 nm. Results are shown as percentages of the control. The morphological changes in neurons were monitored under an inverted phase-contrast microscope. Comparison of means from the absorbance values between differently treated groups was carried out with one-way ANOVA, followed by least significant different test, using SPSS 12.0 software. The value of *P* < 0.05 was considered statistically significant.

### 2.6. Intracellular Free Calcium Concentration ([Ca^2+^]_**i**_) Measurement with Exposure to MA

To determine intracellular free concentrations [Ca^2+^]*_i_*, the neurons, grown for 6 days on glass bottom culture dishes, were incubated with a gradient of MA concentrations (0, 25, 50, 100, and 200 *μ*M) at 37°C for 24 hours. Each concentration's test was performed in triplicate. The cultures were subsequently incubated with calcium sensing Fluo-3/AM at a final concentration of 5 *μ*M for 45 minutes, which then subjected to real-time visualization. Three to five random views of each culture dish for semi-quantitative analyses were acquired under Zeiss LSM 510 laser scanning confocal microscope (Carl Zeiss, Germany). The morphologically normal neuron cells were selected for analysis. The fluorescence intensity values of Fluo-3/AM, that is, [Ca^2+^]*_i_*, were determined with an image analyzing software (Image-Pro Plus, USA). Statistical comparison was performed with one-way ANOVA followed by Dunnett's T3 test, using SPSS software. The value of *P* < 0.05 was considered statistically significant.

### 2.7. Time Course of MA-Induced [Ca^2+^]_*i*_ Changes after 24 Hours Exposure to MA

To determine the effect of Rhy on [Ca^2+^]*_i_*, we performed time course studies in the neurons pretreated with 25 *μ*M MA for 24 hours and measured the difference in [Ca^2+^]*_i_* between cells that received Rhy and the ones that did not. After 6 days' culture, the neurons were incubated with 25 *μ*M MA at 37°C, 5% CO_2_ incubator for 24 hours. The culture was subsequently incubated with 5 *μ*M Fluo-3/AM for 45 minutes. Then the neurons were rinsed twice with serum-free medium containing 25 *μ*M MA. Finally, the cultures were prepared in 25 *μ*M MA medium, subjected to Rhy interference. The cultures were then maintained at 37°C throughout the course. Time course studies for the alteration of [Ca^2+^]*_i_* were as follows: a. MA alone group: neurons in 25 *μ*M MA (0–430 seconds) + medium (no Rhy) (430–860 seconds); b. MA + Rhy group: neurons in 25 *μ*M MA (0–430 seconds) + 25 *μ*M Rhy (430–860 seconds); c. Rhy alone group: normal neurons (0–430 seconds) + 25 *μ*M Rhy (430–860 seconds).

The following experiments were conducted according to Tao-Cheng et al.'s protocols [10]. Briefly, cultures were observed on a laser scanning confocal microscope with 488 nm excitation. Time lapse images were collected in 10-second intervals. Solution changes were prepared by adding fresh solution. We analyzed ten neuronal cells at 0, 430, and 860 seconds. Fluorescent intensity of each neuronal cell body at each time point was obtained with Image Pro Plus. Data were expressed as mean fluorescence intensity values ± SEM. The differences of [Ca^2+^]*_i_* fluorescence between 860 and 430 seconds, 430 and 0 seconds were determined by paired-samples Mann-Whitney *U* test in each group, using SPSS software. Comparison of Fluo-3/AM-indicated [Ca^2+^]*_i_* variances between 430 and 860 seconds of different groups, were performed with one-way ANOVA followed by *post hoc* test. The value of *P* < 0.05 was considered statistically significant.

## 3. Results

### 3.1. MA Showed a Dose-Dependent Neurotoxicity

**Figure 2:**
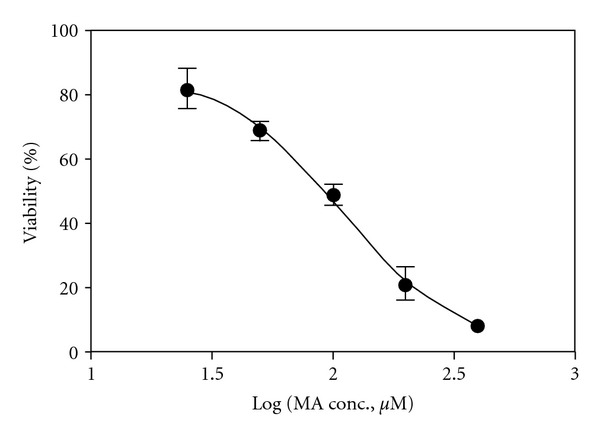
The dose-response curve of neuronal cultures exposed to methamphetamine (MA) determined by MTT assay, which was generated by Prism software. The cortical neurons were exposed to various concentrations of MA (0, 25, 50, 100, 200, and 400 *μ*M) for 48 hours.

The neurotoxic effect of MA was measured with MTT assay, which indicated the function of mitochondrial succinate dehydrogenase. As shown in Figure 2, MA exhibited a dose-dependent neurotoxicity (*R* ^2^ = 0.9568) within the present concentration range. IC_50_ of MA was 111.5 *μ*M (Log IC_50_ SEM: 0.07357 *μ*M).

### 3.2. Morphological Alterations after Exposure to MA

**Figure 3:**
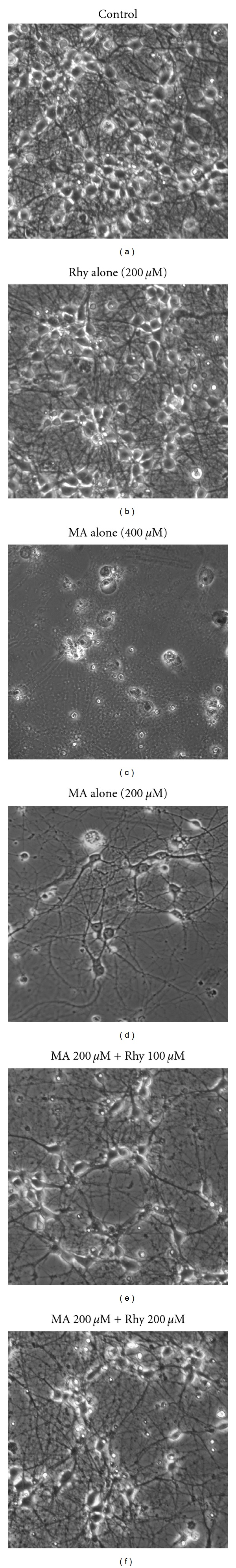
Phase-contrast photomicrographs of control, methamphetamine (MA) and combination cultures after incubation for 48 hours.

Figure 3 shows the representative phase-contrast photomicrographs of control, MA and combination cultures after incubation for 48 hours.

### 3.3. Protective Effect of Rhy on Cortical Neurons after Exposure to MA

**Figure 4:**
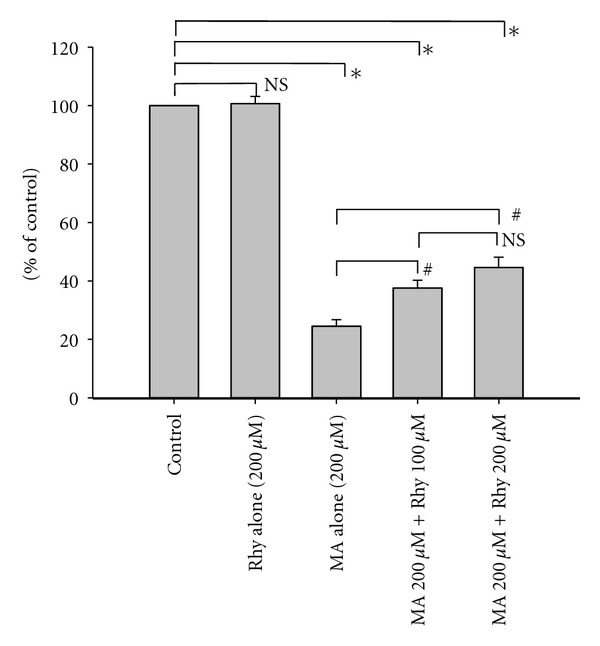
Combination effects of rhynchophylline (Rhy) and methamphetamine (MA) on neocortical cells. Each bar represents the mean percentage of control ± SEM. *P < 0.001  *versus* the untreated control neurons; ^#^ *P* < 0.001  *versus* MA (200 *μ*M) alone group as indicated; NS, not significant.

Incubation with only 200 *μ*M Rhy for 48 hours did not show significant toxicity in the neuronal cultures when compared with untreated cultures. The addition of Rhy 10 minutes prior to the exposure to MA had a significantly positive effect on neuronal survival. Both 100 *μ*M and 200 *μ*M Rhy appeared to have significant neuroprotective effects against 200 *μ*M MA-induced neurotoxicity (*P* < 0.001 in both cases, Figure 4).

### 3.4. Increment of [Ca^2+^]_*i*_ Within Cortical Neurons after Exposure to MA for 24 Hours

**Figure 5:**
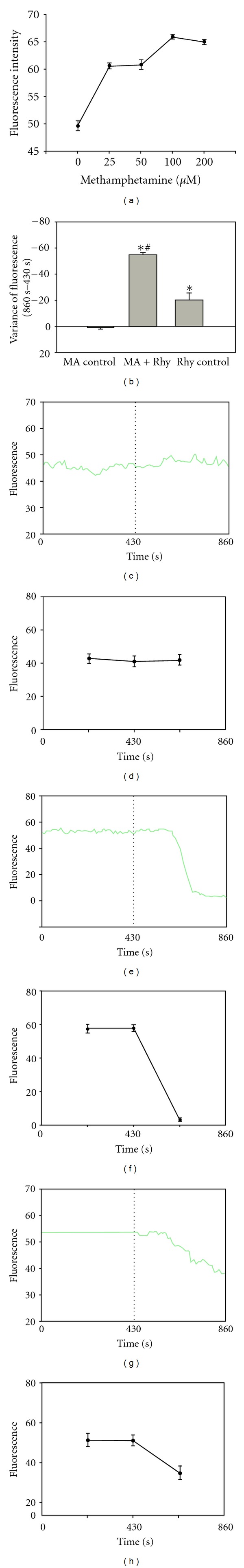
Time course changes of the intracellular free calcium concentration ([Ca^2+^]*_i_*) indicated by Fluo-3/AM. The neurons were exposed to 25 *μ*M methamphetamine (MA) for 24 hours before rhynchophylline (Rhy) intervention. (a) Fluorescence intensity values of Fluo-3/AM, give an indication of [Ca^2+^]*_i_* in neurons 24 hours after exposure to a gradient of MA concentrations (0, 25, 50, 100, and 200 *μ*M) (average value of triplicates). Five images were captured of one culture. Around 150–200 neurons were analyzed in each group. The full dots represent the mean fluorescence intensities ± SEM. *P < 0.001  *versus* normal neurons control without exposure to MA. (b) Fluo-3/AM-indicated [Ca^2+^]*_i_* variation between 430 and 860 seconds in neuronal cells, which was detected with Rhy addition after exposure to 25 *μ*M MA for 24 hours. *P < 0.001  *versus* the MA alone control group. ^#^ *P* < 0.001  *versus* the Rhy alone control group. (c) and (d) MA alone control group: neurons in 25 *μ*M MA + medium (no Rhy). (e) and (f) MA + Rhy group: neurons in 25 *μ*M MA + 25 *μ*M Rhy. (g) and (h) Rhy alone control group: normal neurons + 25 *μ*M Rhy. Each of (c), (e), and (g) was recorded for [Ca^2+^]*_i_*  fluorescence in the representative single neuronal cell. Each of (d), (f), and (h) show the average change of [Ca^2+^]*_i_*  fluorescence in 10 neuronal cells. The full dots represent the mean fluorescence intensity values ± SEM of 10 neurons. *P < 0.001  *versus* the previous time point.

MA induced notable calcium overload in the cortical neurons after exposure to MA for 24 hours. [Ca^2+^]*_i_* was indicated by the fluorescence intensity of Fluo-3/AM excited at 488 nm. As shown in Figure 5(a), MA significantly increased [Ca^2+^]*_i_* at the concentration range from 10 to 150 *μ*M. The mean [Ca^2+^]*_i_* fluorescence values were 60.512 ± 0.530, 60.754 ± 0.860, 65.831 ± 0.448, and 64.941 ± 0.498, after exposure to 25, 50, 100, and 200 *μ*M MA (*P* < 0.001, *versus* control), respectively. The [Ca^2+^]*_i_* was elevated as the exposure to MA concentrations increased. However, MTT assay demonstrated that the number of viable neurons was dramatically decreased after exposure to 200 *μ*M. Thus the intracellular Fluo-3/AM poured out from more dying cells, which still displayed relatively normal morphology but explicating a slightly decreased fluorescent intensity.

### 3.5. Rhy Inhibited Calcium Influx in Neurons after Exposure to MA

One-way ANOVA showed significant difference in the comparison among groups (*P* < 0.001; Figure 5(b)). The mean fluorescence [Ca^2+^]*_i_*  variances between 430 and 860 seconds of the MA alone control group, the MA + Rhy group, and the Rhy alone control group were 0.849 ± 0.834, −54.824 ± 1.767, and −19.722 ± 2.232, respectively. Dunnett's T3 test was used for *post hoc* comparisons, the [Ca^2+^]*_i_* variances of the MA + Rhy group were significantly different from the MA alone control group (*P* < 0.001). The inhibition of the intracellular calcium influx by Rhy alone (Rhy control group) is significantly stronger in neurons after exposure to MA than in untreated normal neurons (*P* < 0.001).

Figures 5(c)–5(h) also shows time course studies of the [Ca^2+^]*_i_* of the cultures exposed to 25 *μ*M MA for 24 hours before Rhy intervention. These results were compared to the effect of the addition of medium (without Rhy) (as reference) on the [Ca^2+^]*_i_*, which showed no change in [Ca^2+^]*_i_* (Figures 5(c) and 5(d)). As seen in Figures 5(e) and 5(f), the paired-samples* t*-tests between 430 and 860 seconds showed that the addition of Rhy at 430 seconds dramatically attenuated the fluorescence intensity of Fluo-3/AM calcium indicators (*P* < 0.001). In the Rhy alone control group, the normal neurons were stimulated with 25 *μ*M Rhy for 430 seconds after normal cell recording. Addition of Rhy alone significantly decreased the fluorescence intensity of intracellular Fluo-3/AM (*P* < 0.01), which is consistent with the calcium channel blocking properties of Rhy (Figures 5(g) and 5(h)).

## 4. Discussion

The majority of researches have been limited to MA neurotoxicity to dopaminergic neurons, in which the dopamine reuptake is inhibited by MA resulting in intracellular high-concentration dopamine [11]. Actually, MA can induce neural impairment in other brain areas too. For example, neurodegeneration has been found in the piriform and parietal cortex, thalamus and hippocampus, areas which receive only a sparse dopaminergic input, and in nondopamine striatal elements [7]. The main objective of the present study is to determine whether the neurotoxic effects of MA as a psychostimulatory drug, which causes neural death on rodent cortical cells *in vitro*, can be attenuated by the pretreatment with Rhy in cell cultures. The neurotoxicity is thought to be linked to intracellular calcium levels, which appear to correlate with MA incubation in a dose-dependent manner. The significant neurotoxicity caused by several types of amphetamines in rat neocortical neurons was previously reported to be accompanied by internucleosomal DNA cleavage and nuclear breakdown, as well as differential expression of the anti- and proapoptotic bcl-xL/S splice variants, indicating an involvement of the apoptotic pathways in amphetamine neurotoxicity [12]. It has been confirmed that MA can be neurotoxic to cortical cells directly, with the increase in calcium load. The [Ca^2+^]*_i_* is therefore suggested to play a pivotal role in MA-induced cell death. MA is a cationic lipophilic molecule that can diffuse into the mitochondria and affects the mitochondrial calcium ATPase, which is responsible for pumping Ca^2+^ into the inner mitochondrial space for storage purposes. The release of these stores, through the mitochondrial membrane that is made permeable by formation of transition pores, triggers the activation of the cell death pathway. Cell death requires the involvement of cysteine proteases and caspases, which are normally associated with the mitochondrial envelope in their inactive state [7]. Consequently, Ca^2+^ influx is followed by mitochondrial damage and reactive species formation. It is well documented that MA induced neurological damage caused by the above described mechanisms, because it is responsible for Ca^2+^ influx due to its effect on ionotropic glutamate receptors [11]. 

Rhy has been proven to be a potent calcium channel antagonist in blood vessels and it is assumed to act as one of the noncompetitive antagonists for glutamate receptors [2]. Therefore Rhy is thought to contribute to the neuroprotective and anticonvulsant activity of plant extracts of *Uncaria* species [13]. Moreover, another *in vivo* behavioral study indicates that Rhy has protective features against harmful effects of MA [14]. To conclude, our study demonstrates that Rhy decreases the high level of intracellular calcium induced by MA in cultured neurons, through the direct blockade of calcium channels and/or the inhibition of ionotropic glutamate receptors.

This is the first report demonstrating an inhibitory effect of Rhy against MA impairment in cultured neurons *in vitro*. Our results suggested that Rhy can protect the neuronal cultures against MA exposure and promptly attenuate intracellular calcium overload triggered by the MA toxin.
